# Acute effects of a single tennis match on passive shoulder rotation range of motion, isometric strength and serve speed in professional tennis players

**DOI:** 10.1371/journal.pone.0215015

**Published:** 2019-04-12

**Authors:** Victor Moreno-Pérez, Álvaro López-Samanes, Raúl Domínguez, Valentín Emilio Fernández-Elías, Pablo González-Frutos, Vicente Fernández-Ruiz, Alberto Pérez-López, Jaime Fernández-Fernández

**Affiliations:** 1 Sports Research Center, Miguel Hernandez University of Elche, Alicante, Spain; 2 School of Physiotherapy, Faculty of Health Sciences, Francisco de Vitoria University, Madrid, Spain; 3 College of Health Sciences, Isabel I University, Burgos, Spain; 4 Physical Activity and Sports, Universidad Europea de Madrid, Madrid, Spain; 5 Departament of Sports Sciences, Faculty of Sports Sciences and Humanities, Francisco de Vitoria University, Madrid, Spain; 6 Department of Biomedical Sciences, Faculty of Medicine and Health Sciences, University of Alcalá, Madrid, Spain; 7 Department of Physical Education and Sports, Faculty of Physical Activity and Sports Sciences, University of Leon, Leon, Spain; Goteborgs Universitet, SWEDEN

## Abstract

Shoulder pain has been associated with glenohumeral internal rotation deficit (GIRD) and a reduction in external rotation (ER) strength; however, in tennis players, there is scarce evidence regarding the impact of a single match on shoulder range of motion (ROM), strength and serve speed. The aim of this study was to determine the acute effect of a single tennis match on shoulder rotation ROM, isometric strength and serve speed. Twenty-six professional tennis players participated in the study (20.4±4.4 years; 10.5±3.2 years tennis expertise; 20.5±5.4 h/week training). Passive shoulder external (ER-ROM) and internal rotation ROM (IR-ROM), ER and IR isometric strength were measured before and after a single tennis match (80.3±21.3 min) in both shoulder´s. Moreover, the total arc of motion (TAM) and ER/IR strength ratio were calculated. Video analysis was used to assess the number of serves and groundstrokes, while a radar gun was utilized to measure maximal ball speed. In the dominant shoulder, compared to pre-match levels, IR-ROM was significantly reduced (-1.3%; *p* = 0.042), while ER-ROM (5.3%; *p* = 0.037) and TAM (3.1%; *p* = 0.050) were significantly increased. In the non-dominant shoulder, ER-ROM (3.7%; *p* = 0.006) was increased. Furthermore, in the dominant shoulder, the isometric ER strength was significantly reduced after the match (-4.8%; *p* = 0.012), whereas serve speed was not significantly reduced after match (-1.16%; *p* = 0.197). A single tennis match leads to significant reductions in shoulder ROM (e.g., IR of the dominant shoulder) and isometric strength (e.g., ER of the dominant shoulder). This study reveals the importance of recovery strategies prescription aiming at minimize post-match alteration in the shoulders.

## Introduction

Competitive tennis requires players to train and compete on a year-round base. This turns into a long season, inability to estimate effectively wins and losses each week at tournaments, high training volumes, and a shifting schedule as ranking changes [1]. As a result of the demands induced by the continuous practice and play, with hundreds of powerful overhead serves and groundstrokes per training/match, tennis players are susceptible to a range of injuries including chronic overuse conditions and acute traumatic injuries [2].

During the repetitive overhead motion of the serve the shoulder is the focal point for force transfer and contributes to 20% of the total force generated during the stroke [3], which, together with its mobility, allowing for a wide range of motion, leads to a fragile balance between stability and mobility when serving [4, 5]. Shoulder injuries prevalence is elevated in tennis players, with an incidence of 8.2 injuries per 1,000 h of play in professional players, and being the 15.9% of all injuries occurred [6, 7]. Consequently, in tennis, the identification of the risk factors for shoulder injury is an essential task to elaborate preventive protocols. Among them, the glenohumeral internal rotation deficit (GIRD) (i.e., > 18–20° reduction in dominant shoulder IR, with a corresponding > 5° loss of total arc of motion (TAM) when compared to the non-dominant shoulder) [8, 9], external/internal (ER/IR) rotation strength ratios (i.e., <60–85%) [8, 10], isometric ER weakness [8, 10], or an external rotation deficiency (ERD) (i.e., > 5° loss in dominant shoulder ER when compared to the non-dominant shoulder) [11], have been highlighted as a risk factors in overhead athletes. However, some conflicting results have been reported regarding these factors. While some authors did not observe an association between GIRD and pain in the dominant shoulder according to players’ levels of expertise (i.e., junior, amateur, elite and professional) [10, 12–14], others reported a significant relationship in amateur [8] and professional players [9]. The methodology used and the moment of the season when shoulders were examined (i.e., in most studies the glenohumeral joint was only examined once at the beginning of the season) are two major confounding variables that may explain this discrepancy [10, 13]. Thus, the in-season physical changes that may precede an injury or even the effects of fatigue after a single tennis match on these musculoskeletal parameters have not been elucidated yet [15].

Only three studies have analyzed glenohumeral joint ROM and force production after a simulated [16] and tennis match [17, 18]. Results showed a reduction in glenohumeral ROM [16–18], isometric rotation strength [17, 19] and serve speed [7, 17] after tennis matches. Nonetheless, in these previous evidences the subjects recruited were young tennis players or non-professional tennis players who were not exposed to the high demands of professional tennis competition [16, 17]. Only Moore-Reed et al. [18] who recruited elite female tennis players, found a reduced IR and TAM after acute exposure to a tennis play.

Therefore, since the shoulder profile (glenohumeral ROM and force production) has not been evaluated after a single competitive match in professional male tennis players, the present study aimed at examining the acute effect of a competitive tennis match on bilateral passive shoulder rotation ROM, isometric ER and IR strength, ER/IR isometric strength ratio and serve speed in the dominant and non-dominant shoulder of professional tennis players.

## Methods

### Ethics statement

The study was reviewed and approved by the scientific and Ethical Review Board of the Francisco de Vitoria University Bioethics Commission approved the study (number 45/2018) and the experimental procedure of this study is in accordance with the latest (7^th^) Declaration of Helsinki and was approved by the Ethics Committee of the University.

### Participants

Twenty-six professional male tennis players (age, 20.38 ± 4.38 years; height, 181.31 ± 8.28 cm; body mass, 72.02 ± 10.17 kg) were recruited from two different high-performance Spanish Academies. At the moment of the study, five players had an Association of Tennis Professionals (ATP) ranking (1262.2 ± 555.9 ATP ranking), six players had an International Tennis Federation (ITF) junior ranking (1169.0 ± 592.0 ITF junior ranking) and fifteen players were among the 300 best Spanish senior tennis players (164.5 ± 71.41). Twenty-three players were right-handed (88.4%) and three were left-handed (11.5%). All tennis players were healthy and competing at the time of the trial (February), trained and average of 11.2 ± 2.7 hours per week of tennis and 5.8 ± 1.1 hours per week of physical training, having a previous average tennis experience of 10.5 ± 3.2 years. The participants were not taking medications throughout the study and they had been free of musculoskeletal injuries during the previous three months.

Before enrolling, participants and parents/guardians (6 participants were under 18 years old) were fully informed before giving their informed written consent. The experimental procedure of this study is in accordance with the latest (7^th^) Declaration of Helsinki and was approved by the Ethics Committee of the University.

### Experimental design

The matches were conducted in two academies during the same month. Upon arrival, participants filled a questionnaire related to sport experience and training background (dominant limb, sports and tennis expertise) and a training regimen questionnaire (weekly practice frequency, hours of tennis practice per week and day); then, the anthropometric measurements were performed. Subsequently, participants carried out a standardized warm-up consisted of 5 min of jogging and 10 min of dynamic stretching exercises [20]. Then, 60 min before the tennis match, the shoulder ROM and strength tests were undergone in a clinical area, after tests and participants’ order randomization [15]. Finally, the serve speed test was performed on the tennis court. Simulated tennis matches were played according to the rules of the ITF, in an outdoor hard-court surface (Greenset surface, GreenSet Worldwide SL, Barcelona, Spain), to the best of a 3-sets system with a super tiebreak in the 3rd set. Matches were paired by players’ level and ranking. Internal and external match load was estimated using the rate of perceived exertion (RPE) scale obtained from each individual player within 30 minutes of completing each match and video camera (Casio Exilim EX-ZR200, Tokyo, Japan), respectively. Five minutes after the tennis match, participants performed the serve speed and shoulders ROM and strength tests in this order. All subjects were instructed to maintain their habitual lifestyle and normal dietary intake before and during the trials and to avoid the ingestion of any stimulant (e.g., caffeine) during the 24 hours before the trial.

### Shoulder ROM test

**Fig 1:**
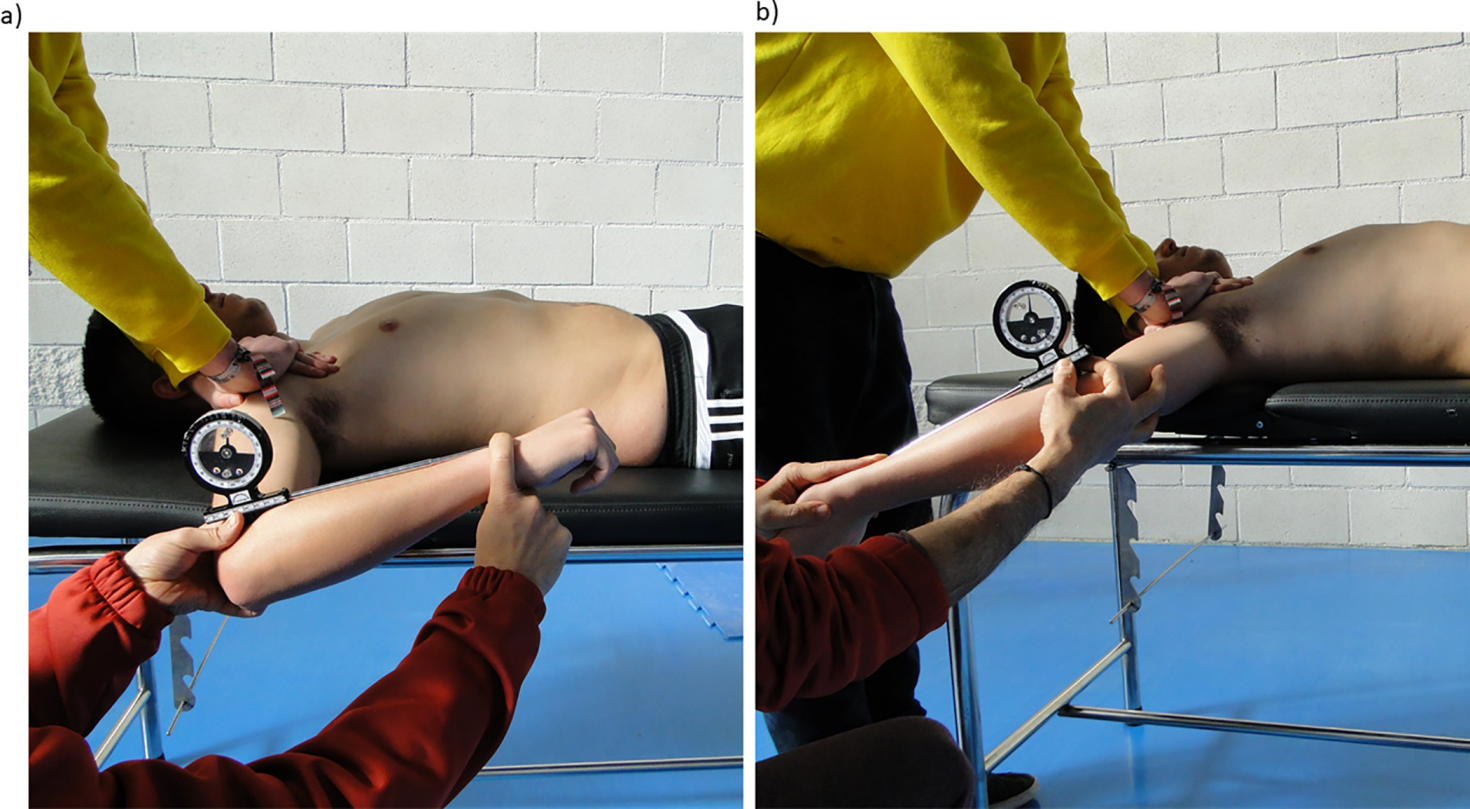
**Assessment of the shoulder rotation range of motion**: a) testing for glenohumeral internal rotation position; b) testing for glenohumeral external rotation position.

Passive shoulder IR and ER ROM were measured with a manual inclinometer (ISOMED inclinometer, Portland, Oregon) in the dominant and non-dominant shoulder [10]. Participants were laid down in supine position on a bench with the shoulder abducted 90° and the elbow flexed at 90° (Fig 1). The inclinometer was placed approximately in the mid-point of the forearm distal end (for IR- and ER-ROM). As starting position, the forearm was pronated and remained in this position during the test. A researcher held participant’s proximal shoulder region (i.e., clavicle and scapula) against the bench to stabilize the scapula while rotating the humerus in the glenohumeral joint to produce maximum passive IR and ER. The end of IR and ER was defined as the point at which the scapula was felt to move [21]. Values (°) for both repetitions (IR and ER) were averaged, and then used to calculate the TAM (the sum of IR and ER ROM). Three trials of each IR- and ER-ROM tests were recorded on each shoulder and the mean score was used for the subsequent analysis.

### Shoulder strength test

**Fig 2:**
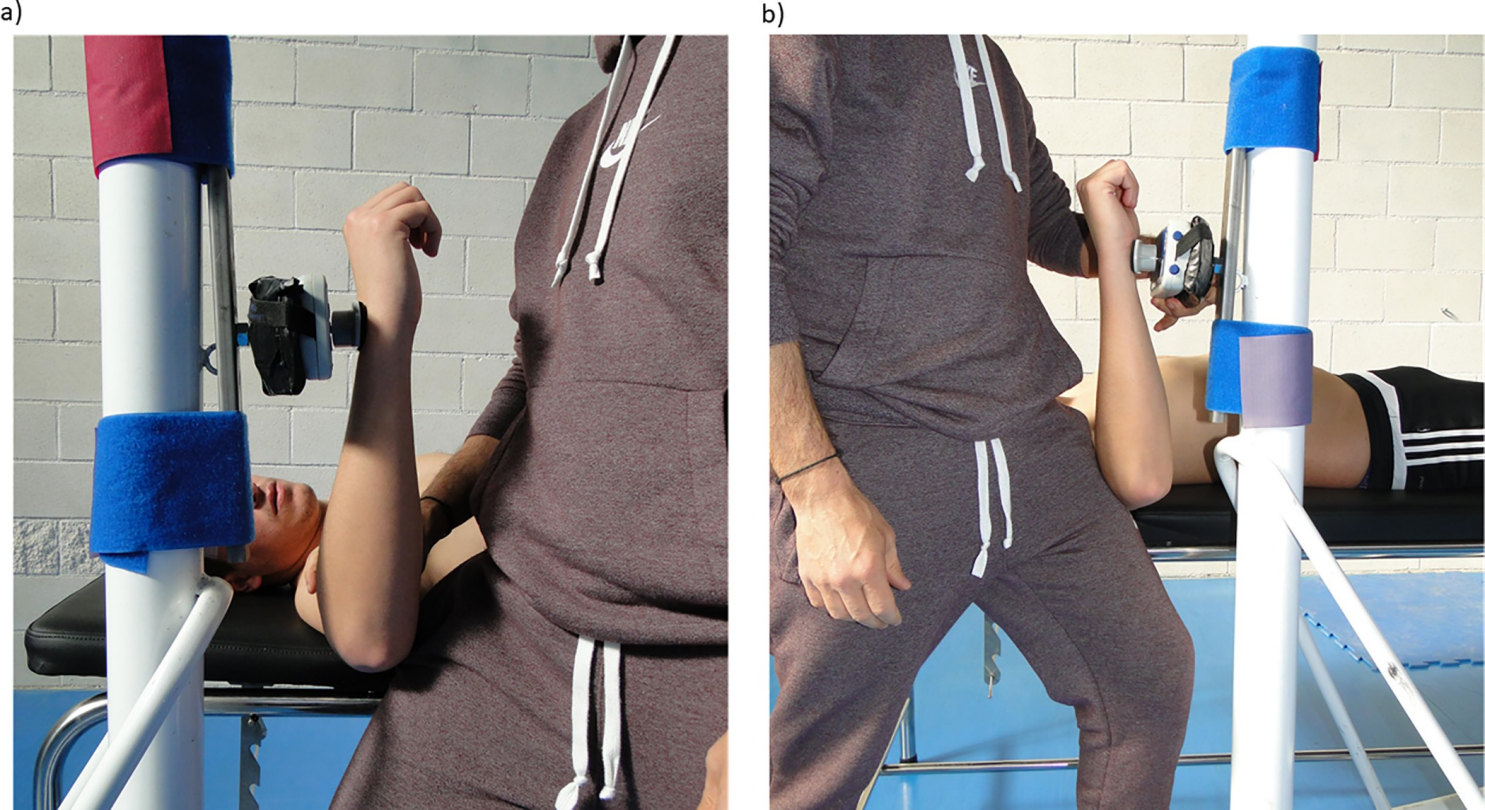
**Assessment of the isometric shoulder rotation strength**: a) testing for shoulder maximal isometric external rotation strength; b) testing for shoulder maximal isometric internal rotation strength.

Shoulder ER and IR strength were measured with a hand-held dynamometer (HHD) (0–500 N range, 0.2 N sensitivity; Nicholas Manual muscle test, Co, Lafayette IN, USA), as previously described elsewhere [10]. The HHD was calibrated according to the manufacturer’s specifications prior to each test. Participants were laid down in supine position on a bench with the arm in 90° of abduction and 0° of rotation, in the scapular plane (Fig 2). The elbow was flexed at 90° with the humerus pressed down toward the bench. The testing angle was visually checked. For ER strength, participants externally rotated the shoulder against the HHD which was located proximal to the ulnar styloid process. For IR strength, participants internally rotated the shoulder against the HHD which was located proximal to the radius styloid process. The dynamometer stability was ensured by fixed the HHD against a stable and plane structure [10]. The isometric strength test for ER and IR consisted of three repetitions of 5 s maximal voluntary contraction with 30 s inter-set rest period. The peak of strength was recorded on the three repetitions for ER and IR tests, and the mean values were calculated by normalizing data using subjects’ body mass.

### Serve velocity

Serve speed was measured by a radar gun (model Pocket Radar Ball Coach PR1000BC, Republic of South Korea), which was set on ‘continuous mode’ to detect maximal ball speed (40 to 210 km/h range). Calibration was performed according to the manufacturer’s specifications prior to each test. The serve test procedure was conducted as previously reported elsewhere [22]. Briefly, the radar was positioned in the tennis court on the center of the baseline, 4 m behind the server, aligned with the approximate height of ball contact (~2.2 m) and pointing down the center of the court. After a brief warm-up consisted of dynamic movements of the shoulder, five second services and five submaximal services, the serve velocity test was performed. Participants were required to serve in a 1 x 1-m area allocated in the farther diagonal corner of the serve area performing five maximal speed serves in as less attempts as possible. The peak velocity of the five serves was calculated for further analysis.

### Internal and external match load

Subjective internal game load of each player was measured using the rate of perceived exertion of each game obtained within 30 minutes after the tennis match. The RPE was then multiplied by the game time of each player, thus reporting the game load in arbitrary units (AU) [23]. Participants were familiarized with the RPE and they were asked to report it confidentially.

All tennis matches were recorded using a diagonally set video camera (Casio Exilim EX-ZR200, Tokyo, Japan) and each point was notated afterward. The number of serves (first and seconds), double faults, aces, forehand, backhand, volleys and smashes were analyzed. Since the matches had a different number of points, the data are presented as absolute values and percentages.

### Statistical analysis

Descriptive statistics (means and standard deviations) for shoulder flexibility (IR- and ER-ROM), shoulder strength (isometric IR and ER), serve speed and internal and external match load variables were calculated. Initially, a Shapiro-Wilk test was used to test the normality of the data (*p* > 0.05) and then a paired *t*-test was run to analyze the differences between the experimental conditions (pre- vs post-match). To verify the pre- vs post-match effect, the effect size (ES) and its 95% confidence interval were calculated [24]. Match analysis variables were correlated (r of Pearson) with ROM, isometric strength and serve velocity. All analyses were performed using the SPSS package (v25, SPSS Inc., Chicago, USA). The results are shown as mean ± SD, and the significance level was set at *p* ≤ 0.05 when the ES 95% confidence interval limits did not cross the zero value.

## Results

### Internal and external match load

The mean duration of the tennis matches was 80.3 ± 21.3 min, environmental conditions were similar (temperature 18.4 ± 6.4°C and humidity 40 ± 8%). The internal match load showed an increase in RPE between pre- vs post-exercise (pre- vs post-match, 3.42 ± 2.08 vs 5.62 ± 2.29, *p* < 0.01). The strokes analysis during the tennis match are showed in S2 Table.

### Shoulder ROM and strength tests

The comparison between pre- vs post-match values of bilateral passive shoulder rotation ROM, isometric rotation strength, and the ER/IR isometric strength ratio are presented in S2 Table.

Compared to pre-match levels, a significantly lower IR-ROM (-1.2%, ES = 0.155, *p* = 0.042), and a significant increase in total ROM (3.3%, ES = -0.318, *p* = 0.05) and ER-ROM (5.7%, ES = -0.549, *p* = 0.037) were found post-match in the dominant shoulder; while, in the non-dominant shoulder a significant increase in ER-ROM was also observed (3.9%, ES = -0.399, *p* = 0.006). Furthermore, participants showed a decreased post-match ER isometric strength in the dominant shoulder compared to pre-match levels (-5.8%, ES = 0.292, *p* = 0.012). No significant differences were observed in the remaining variables analyzed.

## Discussion

The aim of the present study was to analyze the acute effect of a single tennis match on bilateral passive shoulder rotation ROM and isometric strength, as well as serve velocity in professional tennis players. Our results revealed that a tennis match lead to a decrease in the IR of the dominant shoulder together with decreased levels of ER strength, while serve velocity was not significantly altered. These findings highlight the relevance of measuring the shoulder function (e.g., IR/ER ROM and strength) after competitive tennis matches, particularly during high training load periods, in order to preserve shoulders health in competitive tennis players.

Previous studies have suggested that a reduction in shoulder IR-ROM increases the risk of shoulder injuries [8]. Our results showed that a simulated competitive tennis match induces an acute reduction of the IR-ROM (-1.3%), with an increase in the ER-ROM (5.3%) of the dominant shoulder, resulting in an increased TAM (3.1%). In the non-dominant shoulder, an increase in ER-ROM was also reported. These results are in line with previous studies conducted in male [16, 17] and female tennis players [18], which reported a reduction in IR-ROM of the dominant shoulder [16–18] ranging from 4.2 to 20.8%, together with an increase in ER-ROM of the non-dominant shoulder ranging 1.8 to 12.2% [16–18]. However, the magnitude of change revealed here was markedly lower than previous studies. This difference may be attributed to the experimental design performed here, since the tennis match durations (e.g., 80 vs 180 min) and the timing of the post-match measures conducted (e.g., 10 min vs 24h after the match) differs significantly when compared this study with previous ones [16–18].

Although significant, the reported decrease in the dominant shoulder IR-ROM after the match cannot be considered as an indicator of injury risk, since the loss of rotation was markedly lower (-0.76°) than the cut-off values proposed (18–20°) [25]. Hence, the magnitude of IR-ROM change found in this population can be attributed to an exercise adaptation induced by repetitive eccentric contractions produced during the numerous powerful strokes of the tennis match [26]. Nonetheless, five players (19.3% of the sample) presented values of IR loss above 18–20°, thus, it cannot be avoided that the repetitive eccentric contraction that occurs during a match together may contribute to cause passive muscular tension and capsular tightness leading to shoulder injuries [25]. Therefore, shoulder rotation flexibility should be monitored after any competitive tennis match.

Muscle strength and power production of the upper limbs is critical to execute the explosive actions required in tennis (e.g., serve speed, groundstrokes) [27]. In the present study, a significant reduction in the ER muscle strength of the dominant shoulder (-4.8%) was found after a single tennis match. This decline was lower compared to previous studies in which reductions ranging from 6.5% to 9% were reported [17, 19]. In addition to the experimental design (match duration and timing of measurements), the decrease in isometric ER may be attributed to the differences in the population analyzed (i.e., adolescents [17] and young adults [19]). It can be speculated that the decrease in ER muscle strength after a single tennis match may be determined by the fatigue induced in response to high and repetitive loading forces generated by strokes performed, mainly serves and groundstrokes. This together with the difference in tennis training experience of the participants recruited here (12.04 ± 5.53 years) and in previous studies (5.6 ± 1.2 years) (17) may suggest that training experience explains, at least partially, the ER muscle strength differences found. In any case, poor ER strength seems to independently increase the risk of shoulder injury [10]; consequently, early identification and management of impaired ER muscle strength in experimented and non-adapted tennis players after matches may help to reduce the risk of shoulder injury.

In contrast to isometric strength, the serve speed test shows a non-significant decrease after the tennis match. Although these results are similar to those reported by Gescheit et al. [19], two studies have reported a significant reduction in serve speed [16, 17]. Interestingly, in the only study conducted in an adult population of tennis player, a 6.5 km/h decrease in serve speed was found after a 3h tennis match [16], differs from the 1.81 km/h reduction seen in the present study after a 81 min tennis match. The intrinsic requirement of precision in the present study (1 x 1m serve area) compared to the serve speed test protocol performed by Martin et al. [16], may explain the differences, as well as the reduced mean serve speed found. Thus, prolonged tennis match duration (> 3 hours) and a high number of strokes (> 700 groundstrokes) seems to be required to stimulate a certain level of fatigue that induces a significant reduction in serve speed in professional tennis players. However, future studies are needed to further explore the relationship among tennis match duration and the number of strokes with serve velocity and fatigue.

Some limitations of the present study must be acknowledged. First, the only time-point selected to measure passive shoulders rotation ROM, isometric muscle strength and serve speed in response to a single tennis match was immediately post-match, future studies should evaluate these variables using several time-point during the 48 hours after the tennis match. Besides, the accumulated training and match load of the tennis players was not examined before the trials and despite it was similar among participants this variable may influence the results obtained in this type of studies.

## Conclusions

A single tennis match produces a reduction in IR-ROM and an elevation in total and ER-ROM in the dominant shoulder, as well as an increase in ER-ROM in the non-dominant shoulder. Moreover, isometric ER muscle strength was decreased in the dominant shoulder. Given the fact that an alteration in glenohumeral ROM and strength promote an increased injury risk of this joint, the present study highlights the relevance of monitoring the decline of IR-ROM and ER muscle strength in tennis players after competitive matches, to preserve shoulders health and performance status. Therefore, preventive programs which included IR and ER stretching protocols should be included as part of tennis players’ training to reduce the risk of injury in the glenohumeral joint.

## Supporting information

S1 Table Tennis match analysis data. (DOCX)

S2 Table Shoulder ROM and isometric strength comparison between pre- vs post-match (N = 26). (DOCX)
