# Supplementary material for: Acute effects of a single tennis match on passive shoulder rotation range of motion, isometric strength and serve speed in professional tennis players
Source: PLoS One. 2019 Apr 12;14(4):e0215015. doi: 10.1371/journal.pone.0215015 (PMC6461272; doi:10.1371/journal.pone.0215015)
Supplement: S1 Table — (DOCX) [file pone.0215015.s001.docx]

Table 1. Tennis match analysis data.

| **Total Serves** | 48.20 ± 13.45 |
| --- | --- |
| **First Serve** | 29.65 ± 10.24 |
| **Second Serves** | 18.55 ± 7.55 |
| **Ace**  **Double Fault** | 1.50 ± 1.61  4.55 ± 2.91 |
| **Forehand** | 105.17 ± 35.85 |
| **Backhand** | 77.72 ± 29.11 |
| **Forehand volley** | 1.61 ± 1.42 |
| **Backhand volley** | 1.44 ± 1.42 |
| **Smashes** | 1.22 ± 1.31 |

Data are presented as mean ± SD.
