# Supplementary material for: Acute effects of a single tennis match on passive shoulder rotation range of motion, isometric strength and serve speed in professional tennis players
Source: PLoS One. 2019 Apr 12;14(4):e0215015. doi: 10.1371/journal.pone.0215015 (PMC6461272; doi:10.1371/journal.pone.0215015)
Supplement: S2 Table — (DOCX) [file pone.0215015.s002.docx]

Table 2. Shoulder ROM, isometric strength and serve velocity comparison between pre- vs post-match (N = 26).

| **Variables** | **Pre-match** | **Post-match** | ***p*** | **ES [95% CI]** |
| --- | --- | --- | --- | --- |
| **ROM IR D** (º) | 57.67 ± 11.77 | 56.94 ± 11.95 | 0.042 | 0.155 [-0.248 to 0.558]* |
| **ROM IR ND** (º) | 72.55 ± 12.53 | 72.81 ± 7.94 | 0.690 | -0.020 [-0.420 to 0.380] |
| **ROM ER D** (º) | 105.84 ± 10.71 | 111.91 ± 11.16 | 0.037 | -0.549 [-0.983 to -0.116]* |
| **ROM ER ND** (º) | 102.45 ± 9.85 | 106.50 ± 9.43 | 0.006 | -0.399 [-0.817 to 0.019]* |
| **TAM D** (º) | 163.51 ± 16.28 | 168.85 ± 14.22 | 0.050 | -0.318 [-.730 to 0.093] * |
| **TAM ND** (º) | 174.99 ± 15.14 | 179.31 ± 14.73 | 0.111 | -0.276 [-0.685 to 0.132] |
| **Strength IR _REL_ D** (N/kg) | 2.0 ± 0.33 | 1.95 ± 0.30 | 0.466 | 0.156 [-0.246 to 0.560] |
| **Strength IR _REL_ ND** (N/kg) | 1.86 ± 0.32 | 1.78 ± 0.27 | 0.302 | 0.229 [-0.177 to 0.636] |
| **Strength ER _REL_ D** (N/kg) | 1.56 ± 0.28 | 1.47 ± 0.25 | 0.012 | 0.292 [-0.117 to 0.703]* |
| **Strength ER _REL_ ND** (N/kg) | 1.52 ± 0.25 | 1.53 ± 0.20 | 0.939 | -0.014 [-0.415 to 0.386] |
| **Ratio ER/IR _REL_ D** (N/kg) | 0.78 ± 0.14 | 0.76 ± 0.12 | 0.646 | 0.111 [-0.290 to 0.513] |
| **Ratio ER/IR _REL_ ND** (N/kg) | 0.84 ± 0.19 | 0.87 ± 0.15 | 0.515 | -0.162 [-0.566 to 0.241] |
| **Serve velocity (km/h)** | 156.2 ± 16.7 | 154.4 ± 15.7 | 0.197 | -0.108 [-0.229 to 0.578] |

Abbreviations: IR = Internal rotation; ER = External rotation; D = Dominant; ND = Non-dominant; TAM = total arc of motion; REL = relative; ES = Effect size mean [95% CI = confidence interval]. Data are presented as mean ± SD. * *p* ≤ 0.05 compared to pre-match values.
